# 

**Published:** 1968-10

**Authors:** 


					VvH ' ? W
'b i, y
October 1968
The
incorporating the medical journal of the south west
Vol 83 (iii and iv) No 308

				

